# Organizational-Level Moderators Impacting Tobacco-Related Knowledge Change after Tobacco Education Training in Substance Use Treatment Centers

**DOI:** 10.3390/ijerph18147597

**Published:** 2021-07-16

**Authors:** Kathy Le, Tzuan A. Chen, Isabel Martinez Leal, Virmarie Correa-Fernández, Ezemenari M. Obasi, Bryce Kyburz, Teresa Williams, Kathleen Casey, Haleem A. Brown, Daniel P. O’Connor, Lorraine R. Reitzel

**Affiliations:** 1 Long School of Medicine, The University of Texas Health Science Center at San Antonio, San Antonio, TX 78229, USA; lek1@livemail.uthscsa.edu; 2 Department of Psychological, Health & Learning Sciences, The University of Houston, 3657 Cullen Blvd Stephen Power Farish Hall, Houston, TX 77204-5029, USA; tchen3@central.uh.edu (T.A.C.); imarti31@central.uh.edu (I.M.L.); vcorreaf@central.uh.edu (V.C.-F.); emobasi@uh.edu (E.M.O.); hbrown4@uh.edu (H.A.B.); 3 HEALTH Research Institute, The University of Houston, 4849 Calhoun Rd., Houston, TX 77204, USA; 4 Integral Care, 1430 Collier Street, Austin, TX 78704, USA; bryce.kyburz@integralcare.org (B.K.); teresa.williams@integralcare.org (T.W.); kathleen.casey@integralcare.org (K.C.); 5 Department of Health & Human Performance, The University of Houston, 3875 Holman Street, Garrison Gymnasium, Room 104, Houston, TX 77204, USA; dpoconno@central.uh.edu

**Keywords:** tobacco, smoking, implementation, organizational change, training, education, knowledge, substance use

## Abstract

Tobacco use is disproportionately elevated among patients with substance use disorders relative to the general U.S. population. Tobacco interventions are lacking within substance use treatment centers (SUTCs) due to lack of knowledge and training. This study examined knowledge gain and the organizational factors that might moderate knowledge gains following tobacco education training provided to employees (N = 580) within 15 SUTCs that were participating in a tobacco-free workplace program. The number of total annual patient visits, unique annual patient visits, number of full-time employees, and organizational readiness for implementing change (ORIC) as assessed prior to implementation were examined as potential moderators. Results demonstrated significant knowledge gain (*p* < 0.001) after training overall; individually, 13 SUTCs had significant knowledge gain (*p*’s < 0.014). SUTCs with fewer total annual patient visits and fewer full-time employees showed greater knowledge gains. The ORIC total score and all but one of its subscales (Resource Availability) moderated knowledge gain. SUTCs with greater initial Change Efficacy (*p* = 0.029), Valence (*p* = 0.027), and Commitment (*p* < 0.001) had greater knowledge gain than SUTCs with lower scores on these constructs; SUTCs with greater Task Knowledge (*p* < 0.001) regarding requirements for change exhibited less knowledge gain. Understanding the organizational-level factors impacting training effectiveness can inform efforts in organizational change and tobacco control program implementation.

## 1. Introduction

Tobacco use is the leading preventable cause of death, disease, and disability in the United States. It has been linked to cancers of various subtypes (e.g., lung, bladder, mouth, and throat), chronic obstructive lung diseases, and cardiovascular diseases [1]. As of 2019, 14% of the general adult population smoked cigarettes [2]. However, these rates are higher among adults with substance use disorders (SUDs), ranging from 65–85% [3]. Importantly, all types of non-nicotine substance dependence have been associated with nicotine dependence [4]. Not only do individuals with SUDs smoke at higher rates, but they also smoke more heavily and have more difficulty quitting [5]. Research has indicated that patients with SUDs are more likely to die from tobacco-related causes than from their non-nicotine substance use [6]. The disproportionate rates of tobacco use and risk of mortality due to tobacco use among patients with SUDs supports a critical need to address tobacco use amongst this population.

Though patients with SUDs are concerned about their tobacco use and are interested in quitting, there are barriers that hinder these efforts [7,8]. Many substance use treatment centers (SUTCs) do not offer tobacco interventions. For example, a study based in California—a state where smoking rates are relatively low and attention to tobacco control relatively high—reported that only about 30–40% of SUTCs offer cessation counseling and only 26% offer cessation medications [9]. One reason for this is that employees within SUTCs often do not feel confident nor feel that they have the skills to treat tobacco dependence [10,11]. Employees within SUTCs often receive little training on empirically-based tobacco control or tobacco use disorder interventions, and a lack of knowledge and training are among the most commonly cited barriers to the provision of tobacco interventions and tobacco-free policy implementation across various addiction treatment settings [6,7,11]. Additionally, although smoking cessation during SUD treatment has been associated with improved outcomes in the treatment of non-nicotine substances, clinicians often hold the misconception that smoking cessation will interfere with successful SUD treatment [12]. Other common clinical misconceptions include that tobacco cessation would be too stressful for patients during SUD treatment and that patients are not interested in quitting smoking [13].

Recently, organizational tobacco control programs have been successful in addressing tobacco use in SUTCs, such as in Texas, New Jersey, New York, Oregon, and Utah [14,15,16,17,18,19]. Employee training has been a major component in many of these multifaceted tobacco control programs because all employees are responsible for the implementation and sustainment of tobacco control policies (e.g., tobacco-free workplaces); however, training has also been provided as a stand-alone effort to promote tobacco intervention provision for patients with SUDs [20,21]. The receipt of training on empirically-based methods to address tobacco use disorders has been associated with an increase in the provision of interventions for tobacco-using SUD patients [21,22]. Among SUTCs that have provided tobacco training for their employees, there have been increases in skills, knowledge, confidence, and supportive attitudes regarding concurrent tobacco treatment with SUD treatment as well as decreases in clinical misconceptions [7,23]. Research suggests that increasing knowledge among clinicians is integral to their implementation of tobacco cessation services: when clinicians had more training and were equipped with tobacco treatment skills, they were more likely to implement the five A’s (ask patients about their tobacco use, advise them to quit, assess their willingness to quit, assist them in quitting, and arrange for follow-up); counsel on tobacco cessation; and provide pharmacotherapy to patients when indicated [24,25]. Additionally, positive attitudes toward tobacco treatment have been associated with increased integration of tobacco treatment within the standard-of-care for the treatment of non-nicotine substance use disorders [14,26].

The importance of employee training is exemplified not only in its association with increased tobacco intervention provision, but also its longstanding status as a major driver of clinician behavior change within implementation science [27]. With this in mind, it is of critical importance to stakeholders, such as policymakers and organizational leadership (e.g., center leadership), to understand organizational factors that might affect training effectiveness. Training effectiveness is typically assessed via attendees’ knowledge change/gain from pre- to post-training [23,27]. Some organizational factors affecting knowledge change after training have been delineated for behavioral health treatment facilities [28]. For instance, perceived value of the change and total number of patient contacts have been seen to moderate training effectiveness among clinicians in behavioral health treatment facilities whereas knowledge of the change and perceived availability of resources have been seen to moderate training effectiveness among non-patient-facing employees [28]. However, how similar organizational factors moderate training effectiveness in SUTCs has yet to be examined. Because there are major differences between non-profit behavioral health treatment facilities and SUTCs, such as size and capacity [25,29,30,31], it is important to delineate how organizational factors affect knowledge change within SUTCs specifically. In addition to providing the organizational context necessary for stakeholders to anticipate the impact of training, this research may also help further understanding on how organizational factors may affect program implementation overall in similar settings. Identification of facilitators to training-related knowledge gain, and by relation, employees’ behaviors and supportive attitudes toward tobacco control organizational policies and practices, is especially important in the context of stagnated translation of evidence-based practices into SUTCs and critical to reducing the research to practice gap [32].

## 2. Materials and Methods

### 2.1. Study Aims, Context, and Hypotheses

The aim of this study was to evaluate knowledge change after provision of tobacco education training to employees at SUTCs and the organizational-level factors impacting knowledge change. Data from the Taking Texas Tobacco Free (TTTF) program were used for these analyses. TTTF is an academic-community partnership that implements a multicomponent, evidence-based tobacco-free workplace program within SUTCs. Through tobacco-free workplace policy development assistance, tobacco education training to all employees, specialized clinical training, resource provision (e.g., nicotine replacement therapy (NRT)), and technical assistance, the TTTF program seeks to promote tobacco treatment for patients with SUDs. We hypothesized that tobacco education training would facilitate an increase in knowledge among employees at SUTCs and that organizational-level characteristics, such as number of patient contacts and pre-implementation organizational readiness for change, would moderate training effectiveness as measured by knowledge gain.

### 2.2. Participating SUTCs, Employees, and Their Reach

From December 2017–May 2020, a total of 19 non-profit SUTCs were enrolled in the TTTF program. SUTCs were recruited primarily through direct email solicitation to the CEO and/or via word of mouth from other SUTCs. Overall, 2 SUTCs withdrew from TTTF program implementation prior to participating in the tobacco education training that is the subject of this study; 1 SUTC withdrew shortly after participating in tobacco education training and completing pre- and post-training knowledge tests; and 1 SUTC withdrew from TTTF program implementation after participating in tobacco education training though did not complete pre- and post-training knowledge tests. Reasons cited for withdrawal by 2 SUTCs were competing demands and shifting priorities due to the pandemic, and the other 2 SUTCs voiced concerns that implementing a completely tobacco-free workplace policy would affect their patient census. The team provided those centers with testimonials and empirical data that countered the “reduced census” fear but to no effect. These aforementioned 4 SUTCs were not included in this study.

Participating SUTCs reported 850 employees (which includes clinicians and non-patient-facing, general employees; see Table 1) altogether. While there were 850 employees reported, only 580 employees took the pre-training knowledge test and 525 employees took the post-training knowledge test. Together, the SUTCs served 82,927 unique patients through 299,267 annual contacts, or total annual patient visits, as per their recent annual reports (see Table 1). Many of these SUTCs served unique populations, including patients who are involved with the criminal justice system, who were vulnerably housed or experiencing homelessness, and who were pregnant.

### 2.3. Procedures

The IRB at the University of Houston approved all study procedures (STUDY00000472, approval date 27 July 2017). The SUTC CEOs or their designees agreed to program participation by signing a Memorandum of Understanding, which detailed overall program requirements and responsibilities. Thereafter, the full TTTF Program was implemented therein over a 7.2–13.6-month period (mean = 10.96, SD = 3.84), which started with ~1–2 h tobacco education trainings that were targeted at all SUTC employees (both clinicians and non-patient-facing employees). Trainings were provided in person prior to the COVID-19 pandemic (which was declared a pandemic on 11 March, 2020 by the WHO) (SUTC 1–12) and virtually thereafter (SUTC 13–15) [33]. The training included education on the dangers of tobacco use, the addictiveness of nicotine, the prevalence of tobacco use among individuals with non-nicotine substance use disorders, the tobacco industries’ targeting of individuals with behavioral health needs, and empirically-based methods to address tobacco use disorder (including the importance of tobacco-free workplace policies and how to uphold them). Several other components of the TTTF program were enacted thereafter as detailed elsewhere [14,28,29,34,35,36,37,38], including sending program champions to Certified Tobacco Treatment Specialist trainings, Motivational Interviewing trainings, etc., but are not the subject of this report. Program champions are individuals who are identified by the center to lead and be the primary contact for implementation of the tobacco-free workplace program within their center. The program champion coordinates the TTTF training, forwards links to employees for TTTF survey completion, monitors NRT distribution, and reports quantities of tobacco screenings and NRT provided.

### 2.4. Measures

#### 2.4.1. Organizational Demographics

The SUTC CEOs or their designees provided information on the number of total annual patient visits, number of unique annual patient visits, and the number of full-time employees prior to TTTF implementation via an online survey.

#### 2.4.2. Organizational Readiness for Implementing Change (ORIC)

The SUTC CEOs or their designees were administered the ORIC [39] within the aforementioned electronic survey, which was administered pre-program implementation. The ORIC has 24 items that comprise a total score (α = 0.73) and 5 subscale scores: (1) perceived availability of resources (Resource Availability), “We have the skills to implement this change,” α = 0.61; (2) organizational efficacy toward change (Change Efficacy), “People who work here feel confident that the organization can get staff invested in implementing this change,” α = 0.93; (3) perceived valence in the change (Change Valance), “We value this change,” α = 0.00 (alpha reflects almost no variation in responses); (4) commitment to change (Change Commitment), “People who work here want to implement this change,” α = 0.86; and (5) knowledge of the requirements for change (Task Knowledge), “We know how much time it will take to implement this change,” α = 0.66. ORIC subscale mean scores and total ORIC mean scores ranged from 1–5; these mean scores were used in analyses. Higher ORIC scores are associated with greater readiness for organizational change [40].

#### 2.4.3. Tobacco Training Knowledge Test

An investigator-generated, 10-item knowledge test was used to assess knowledge gain, which was administered before and after the ~1–2 h TTTF-led tobacco training. Items were face-valid and directly reflected training content. They included “Which of the following is not one of the ‘Five A’s’ of tobacco cessation brief intervention” (response options = Ask, Arrange, Assess, and Allow) and “Which of these tobacco treatment medications requires a prescription?” (response options = nicotine patch, nicotine inhaler, nicotine lozenge, nicotine gum, and all of the above). These tests were administered anonymously; consequently, pre- and post-tests could only be matched to SUTC. Moreover, the pre- and post-training knowledge test sample sizes may differ within SUTCs based on compliance with requests to take the pre- or post-tests, having to leave during the training for job duties/emergencies prior to post-test administration, etc. Possible scores for the knowledge test ranged from 0 to 10 for both the pre- and post-training knowledge test; higher scores indicated greater knowledge.

### 2.5. Statistical Analysis

Tobacco-related knowledge change was assessed for each SUTC separately and for all SUTCs combined. Independent t-tests were conducted to examine the pre-training and post-training knowledge change as the data were un-matched at the participant level. Cohen’s *d* was calculated to assess effect size. The potential moderating effects of organizational demographics (i.e., number of total annual patient visits, number of unique annual patient visits, and number of full-time employees) and readiness to change (via the ORIC total and subscales) on knowledge change over time were examined with interaction terms. Continuous variables were mean-centered prior to moderation analyses. Linear mixed models were performed to account for the nested data structure of participants within the SUTC. All analyses were conducted using SAS 9.4. Alpha was set at 0.05.

## 3. Results

On average, the 15 SUTCs included in the analyses reported 19,951.13 total annual patient visits (SD = 29,520.62, observed range = 535–101,869); 5528.47 unique annual patient visits (SD = 16,473.65, observed range = 45–64,419); and 56.67 full-time employees (SD = 82.47, observed range = 5–304). The means (±SD) of the ORIC were as follows: Resource Availability (4.25 ± 0.53), Change Efficacy (4.85 ± 0.31), Change Valence (4.99 ± 0.05), Change Commitment (4.71 ± 0.37), Task Knowledge (4.24 ± 0.58), and total ORIC (4.67 ± 0.19).

### 3.1. Pre- to Post-Test Knowledge Change

The overall average score was 5.57 (*SE* = 0.07) on the pre-test and 7.44 (*SE* = 0.08) on the post-test, both out of a possible 10.0. The knowledge test score changes (the number of correct items from pre- to post-training) ranged from 1.2 to 4.0 (Table 2). Overall, the gain in tobacco-related knowledge in SUTCs following tobacco education training was statistically significant (change = 1.87, *p* < 0.001), suggesting training effectiveness. Of the 15 SUTCs, employees from 13 showed significant knowledge gain (pre-test: 4.58 to 6.20; post-test: 6.77 to 9.29); employees from two SUTCs (i.e., SUTC 3 and SUTC 9) demonstrated knowledge increases that were not significant but were amongst the top two highest scoring SUTCs on the pre-test. A large effect for score change from pre- to post-training was observed for each participating SUTC (Table 2).

### 3.2. Knowledge Gain Moderators: Organizational Demographics

The number of total annual patient visits (*b* = −0.000006, *SE* = 0.000002, *p* = 0.008) and number of full-time employees (*b* = −0.004, *SE* = 0.0009, *p* < 0.001) were significant moderators of training effectiveness (Table 3). The significant interaction indicated that SUTCs with fewer total annual patient visits reported greater knowledge gain from pre-training to post-training relative to SUTCs with more total annual patient visits. Additionally, SUTCs that had fewer full-time employees demonstrated greater knowledge improvement from pre-training to post-training compared to SUTCs with more full-time employees.

### 3.3. Knowledge Gain Moderators: Organizational Readiness

The ORIC moderated training effectiveness (*b* = 0.902, *SE* = 2.904, *p* = 0.004). SUTCs with higher ORIC scores demonstrated greater knowledge gain from pre- to post-training. Specifically, all ORIC subscales except Resource Availability were significant moderators of knowledge gained from pre- to post-training: Change Efficacy (*b* = 0.669, *SE* = 0.306, *p* = 0.029), Change Valence (*b* = 2.810, *SE* = 1.267, *p* = 0.027), Change Commitment (*b* = 1.224, *SE* = 0.320, *p* < 0.001), and Task Knowledge (*b* = −0.987, *SE* = 0.268, *p* < 0.001) were significant moderators of knowledge gain over time (Table 3). Examination of the significant moderation effects showed that the SUTCs with greater knowledge of requirements for change exhibited less knowledge gain. However, SUTCs with greater initial efficacy, valence, and commitment reported greater knowledge gain than those SUTCs with lower scores on these constructs.

## 4. Discussion

Currently, there is a dearth of literature that details the factors impacting organizational change and educational program implementation among SUTCs, where there especially is a need for translational research and a reduction of the research to practice gap regarding addressing tobacco use disorder amongst patients with SUDs [32]. In this study, we evaluated training effectiveness (SUTC employee knowledge gain from before to after tobacco education training provision) and the organizational-level factors that moderated training effectiveness. Results demonstrated that, overall, SUTC employees increased tobacco-related knowledge from pre- to post-training. Thus, tobacco education training was an effective mechanism to increase knowledge gain, a commonly cited barrier to tobacco intervention [7]. Additionally, we identified organizational-level factors including center demographics and readiness for change that moderated the training effectiveness. Regarding center demographics, centers with fewer numbers of total annual patient visits and fewer numbers of full-time employees had greater knowledge gain. Regarding center readiness, centers with greater readiness, as indicated by higher ORIC total scores, had greater knowledge gain. Additionally, all ORIC subscales apart from Resource Availability (i.e., Change Efficacy, Change Valence, Change Commitment, Task Knowledge) moderated knowledge gain. These results highlight the role of training in increasing tobacco-related knowledge (e.g., about tobacco interventions, tobacco harms and risks) among SUTC employees in Texas. These results also elucidate organizational factors that can facilitate or hinder this knowledge change, addressing a current gap in the literature and allowing comparison with a prior study conducted within larger and better-resourced behavioral health treatment facilities [28].

The overall improvement in tobacco-related knowledge among SUTCs suggests that tobacco education training was an effective method to increase SUTC employee knowledge on the harms of tobacco use, the addictiveness of nicotine, the prevalence of tobacco use among individuals with non-nicotine substance use disorders, the tobacco industries’ targeting of individuals with behavioral health needs, tobacco-free workplace policy implementation, and available tobacco cessation interventions. Among the 15 participating SUTCs, 13 displayed significant knowledge gain among employees; all 15 SUTCs demonstrated improvement and large effect sizes. The two SUTCs that did not have significant knowledge gain had scored highly on the pre-training assessment, suggesting that a lack of significant improvement may have been due to an already elevated level of baseline tobacco-related knowledge at these centers. The significant knowledge gains demonstrated overall are consistent with prior research conducted within behavioral health treatment facilities in Texas, which demonstrated significant knowledge gain after tobacco education training for both clinicians and non-patient-facing employees [28]. Taken together, these results suggest that tobacco education training is needed to address employee knowledge gaps in tobacco control in treatment settings that serve individuals with co-occurring mental and substance use disorder issues. Moreover, the 1–2 h trainings were feasible for the participating SUTCs, which suggests that for lower resourced organizations, such as SUTCs, training can not only be organized but is also effective in increasing tobacco-related knowledge. Other prior work suggests that training improves attitudes about tobacco intervention; increases confidence and willingness to treat tobacco; and increases belief that employees have a role to play in tobacco control in the context of treating SUDs [23]. Ultimately, these factors likely contribute to whether or not clinicians provide tobacco interventions for patients and may affect all employees’ willingness to implement and uphold a tobacco-free workplace policy [23,25,37,41,42,43].

Center demographics—specifically, total annual patient visits and number of full-time employees—moderated training effectiveness. SUTCs with fewer total annual patient visits and fewer full-time employees evinced greater employee changes in knowledge from pre- to post-training. This may suggest that something about smaller center size is a facilitator of knowledge gain. This is counterintuitive, however, as some research suggests larger organizations have more resources and infrastructure to enact tobacco control changes and thus are generally more successful in their implementation [44,45]. However, this pattern of results is consistent with our prior work in behavioral health treatment facilities in Texas [28], suggesting that seemingly low-capacity treatment centers will especially benefit from employee tobacco education trainings. This interpretation is further supported by the fact that Resource Availability, a subscale of the ORIC, was not a moderator of knowledge gain in our study. One possible explanation why knowledge gain was greater in smaller centers is that a small center might have a more conducive environment to foster stronger relationships and communication between an organization’s management and its employees. Additionally, in smaller centers, center leadership may be able to work more closely with employees to iterate the importance of tobacco control within the context of non-nicotine substance dependence treatment and/or they can more easily ensure all employees are aware of the importance of this mission [14]. Together, these factors have been shown to facilitate organizational change [42,46] and may foster greater receptivity to training within the context of a tobacco-free workplace program implementation. Because one possible mechanism through which smaller center size confers advantage to implementation is through stronger relationships and communication between employees and management, smaller SUTCs might consider ways to use these conditions to enhance buy-in amongst employees for the implementation of these educational initiatives and comprehensive tobacco-free workplace programs such as TTTF.

Organizational readiness for change is often important to successful initiative and program implementation [47]. SUTCs with higher ORIC scores demonstrated greater knowledge gain. This is consistent with prior research indicating that when organizational readiness for change is high, leadership and employees are more likely to enact the change, dedicate greater effort towards the change, be more persistent, and display more cooperative behavior [47]. It is possible that SUTCs with higher ORIC scores had employees who were more dedicated to the implementation of the TTTF program. This may have made employees more cooperative with the training, thereby facilitating training effectiveness. Alternatively, high organizational readiness for change may correlate with a greater acceptance of a need for change and buy-in for the change, facilitating employee openness to learning more about empirically-based tobacco control policies and practices [48]. In fact, the ORIC subscale moderator analyses in this study supported greater pre- to post-training knowledge gain amongst SUTCs where center leadership believed their employees could take the steps needed for change (Change Efficacy); cared about/valued the change (Change Valence); and were motivated to implement the change (Change Commitment). Prior research supports that when the reason for the organizational change, or its value, is clearly understood by employees (e.g., health benefits to patients), there is greater likelihood of engagement in the process [46,49]. Consequently, among SUTCs that had greater Change Valence, employees may have been more encouraged to understand the material in order to have the knowledge to provide patients with tobacco interventions, which they considered valuable and beneficial for their patients. This is in line with motivation theory that suggests individuals are more likely partake in activities where there are perceived rewards [48]. Consequently, in SUTCs where there is low Change Valence, employees might be more resistant to change and less engaged with training [46]. More research is needed to understand the dynamics underlying these results; however, findings suggest the potential value in centralized communication from leadership to their employees regarding the rationale for the change given that employees’ understanding of the need for change and its value can facilitate organizational change success. Ensuring that SUTC leadership and management staff are well-informed of the problem of tobacco use amongst their patient stakeholders and the benefits of comprehensive tobacco-free workplace programming (though education provision) seems advisable to promote supportive messaging to SUTC employees.

Interestingly, SUTCs with greater knowledge about the requirements for change (higher ORIC Task Knowledge scores) demonstrated less knowledge improvement from the provided training. The opposite pattern of results was found in our prior study with behavioral health treatment facilities; however, only among the non-patient facing employees [28]. Corresponding moderator analyses with clinicians were non-significant in that study [28]. In this study, SUTCs with greater employee Task Knowledge generally started with greater knowledge of the training content (i.e., higher pre-test knowledge scores). While this association was not significant (*r* = 0.02, *p* = 0.57), it suggests that one reason for the lower knowledge gain was an elevated baseline level of tobacco-related knowledge. Consequently, this suggests that to promote greater knowledge gain, SUTCs should seek to tailor their training to target areas that have not yet been mastered by their employees and would represent relevant areas of improvement for them. This may build the breadth of employees’ knowledge and, in turn, positively impact the organizational change.

This study has limitations. First, our data comprise centers that were able to implement the TTTF program and may not necessarily reflect the characteristics of centers that are more likely to drop out or less likely to enroll in the program. Second, while our research presents factors that moderate training effectiveness, it does not discuss the steps a SUTC should take to address their organization’s readiness for change in a way that facilitates training effectiveness. Consequently, future research should look into effective mechanisms for organizations to improve likelihood of successful organizational change. Third, we did not match pre–post test results by participants. That is, to keep participants anonymous, we were only able to compare pre- and post-test results by SUTC rather than at the individual level. Due to this design, there were circumstances whereby some participants completed a pre-test but not a post-test. Additionally, we could not separate results between clinicians and non-patient-facing employees to compare knowledge gain as we did in a prior study [28]. Consequently, future research might consider matching training results by participant to assess individual knowledge change as well as comparing knowledge change between clinicians and non-patient-facing employees. Fourth, our study design did not include assessment of the maintenance of knowledge gains over time, which may be important in clinician and employee behavior changes in tobacco control implementation. Lastly, the ORIC took into account only the perspectives of SUTC CEOs or their designees. While questions asked the CEO to assume the position of their employees (“The people who work here…” or “We believe…”), it would be beneficial to include employees themselves in assessments regarding center readiness for change in future research.

## 5. Conclusions

This study contributes to the groundwork of implementation science among SUTCs and supports the role of a brief (~1–2 h) training in increasing tobacco-related knowledge among SUTC employees. Practically speaking, results suggest that employees of SUTCs can benefit from tobacco-related trainings, which may be an essential stepping stone to better addressing tobacco use amongst their patient stakeholders. Moreover, SUTCs with fewer total annual patient visits and fewer full-time employees had greater knowledge gain from training, suggesting that not only is knowledge gain significant and feasible in smaller-sized SUTCs after a tobacco education training but also that smaller SUTCs seem to have a slight advantage in knowledge gain relative to larger SUTCs. Consequently, leadership from small SUTCs, which may be lesser resourced than larger SUTCs, should not be deterred from implementing similar training programs, as these efforts may yield substantial knowledge gains. There are many resources to aid in this training provision, including live or recorded webinars offered by groups including the University of California—San Francisco’s Smoking Cessation Leadership Center, the National Council for Mental Wellbeing (formerly the National Council for Behavioral Health), the American Lung Association, and Taking Texas Tobacco Free, to name a few. Additionally, as greater organizational readiness for change was largely associated with greater knowledge gain, the implementation of comprehensive tobacco-free workplace programs that include similar educational efforts would benefit from ensuring that SUTC leadership clearly communicate their enthusiasm and perceived organizational capacity for making this change to their employees. Overall, these results on how center size and center readiness for change impact training effectiveness can help inform policymakers and organizational leadership as they pursue quality improvement programs and similar organizational-level changes. However, future work is still needed to study mechanisms through which these organizational-level factors moderate training effectiveness in both the short- and long-term.

## Figures and Tables

**Table 1:** Participating substance use treatment centers, employees, and their reach.

Center	Total Annual Patient Visits	Unique Annual Patient Visits	Full-time Employees	Implementation Dates	Unique Populations Served
SUTC 1	800	385	22	8 December 2017–4 December 18	98% CJS 15% H 1% SM 8% P
SUTC 2	1004	1004	165	19 January 2018–15 August 2019	70% CJS 68% H
SUTC 3	535	377	6	15 February 2018–29 November 2018	65% CJS 10% H 1% SM
SUTC 4	15,500	2000	85	15 February 2018–17 October 2018	10% CJS 15% H 80% SM <1% P
SUTC 5	64,419	64,419	104	2 May 2018–10 June 2019	Information not available.
SUTC 6	2052	1216	55	18 December 2018–27 January 2020	35% CJS 12.5% H 32% P
SUTC 7	15,572	199	15	13 June 2019–18 February 2020	30% CJS 2% H 3% P
SUTC 8	2000	1800	19	16 August 2019–28 September 2020	20% H
SUTC 9	3521	45	10	5 September 2019–24 June 2020	45% CJS 10% H 1% SM
SUTC 10	13,300	170	7	6 September 2019–04/09/2020	15% CJS 7% H 2% SM 3% P
SUTC 11	7825	100	9	1 October 2019–24 June 2020	5% CJS 20% H 5% SM 15% P
SUTC 12	50,000	350	9	8 October 2019–24 June 2020	35% CJS 15% H 5% SM 3% P
SUTC 13	101,869	9856	304	12 December 2019–28 September 2020	13% CJS 10% H 1% SM 1% P
SUTC 14	20,000	256	5	19 May 2020–4 January 2021	5% CJS 5% H 15% SM 1% P
SUTC 15	870	750	35	24 May 2020–4 January 2021	100% CJS 20% H <10% SM <2% P

Note: SUTC = substance use treatment center. CJS = patients who are engaged in the criminal justice system in some way (e.g., on parole or enrolled in an SUTC as a jail diversion or alternative to jail). H = patients that have been homeless in the past 5 years or at high risk for future homelessness. SM = patients who identify as sexual minorities. P = patients who are pregnant.

**Table 2:** Pre- and post-training knowledge test scores and change (N = 580/525 employees).

Center	Pre-Test N	Pre-Test Mean (95% CI)	Pre-Test SE	Post-Test N	Post-Test Mean (95% CI)	Post-Test SE	Score Change (95% CI)	*p*-Value	Cohen’s d
All	580	5.57 (5.43, 5.72)	0.07	525	7.44 (7.29, 7.59)	0.08	1.87 (1.66, 2.08)	<0.001	1.00
SUTC 1	12	4.58 (3.22, 5.95)	0.62	15	7.53 (6.78, 8.28)	0.35	2.95 (1.55, 4.35)	<0.001	1.60
SUTC 2	4	4.75 (2.36, 7.14)	0.75	4	8.75 (7.23, 10.27)	0.48	4.00 (1.82, 6.18)	0.004	2.55
SUTC 3	5	6.00 (4.48, 7.52)	0.55	5	7.20 (4.51, 9.89)	0.97	1.20 (−1.37, 3.77)	0.313	0.65
SUTC 4	66	6.00 (5.58, 6.42)	0.21	58	8.33 (7.88, 8.78)	0.22	2.33 (1.71, 2.94)	<0.001	1.25
SUTC 5	42	5.21 (4.71, 5.72)	0.25	41	7.56 (7.12, 8,00)	0.22	2.35 (1.68, 3.01)	<0.001	1.35
SUTC 6	29	5.21 (4.61, 5.80)	0.29	27	8.33 (7.68, 8.99)	0.32	3.13 (2.26, 3.99)	<0.001	1.74
SUTC 7	12	5.50 (4.24, 6.76)	0.57	12	8.33 (7.55, 9.12)	0.36	2.83 (1.44, 4.23)	<0.001	1.58
SUTC 8	14	5.14 (4.40, 5.89)	0.35	13	7.38 (6.29, 8.48)	0.5	2.24 (1.00, 3.48)	0.001	1.28
SUTC 9	5	6.20 (4.36, 8.04)	0.66	5	7.40 (5.98, 8.82)	0.51	1.20 (−0.73, 3.13)	0.189	0.74
SUTC 10	8	5.63 (4.22, 7.03)	0.6	5	8.20 (7.16, 9.24)	0.37	2.58 (0.77, 4.38)	0.009	1.55
SUTC 11	124	5.35 (5.02, 5.68)	0.17	110	6.77 (6.40, 7.15)	0.19	1.43 (0.93, 1.92)	<0.001	0.73
SUTC 12	8	5.00 (3.33, 6.67)	0.71	6	8.00 (6.12, 9.88)	0.73	3.00 (0.75, 5.25)	0.013	1.53
SUTC 13	221	5.81 (5.59, 6.04)	0.11	207	7.26 (7.04, 7.47)	0.11	1.44 (1.13, 1.75)	<0.001	0.80
SUTC 14	9	5.56 (3.83, 7.28)	0.75	7	9.29 (8.41, 10.17)	0.36	3.73 (1.78, 5.68)	0.001	2.03
SUTC 15	21	5.24 (4.48, 6.00)	0.36	10	7.10 (6.31, 7.89)	0.35	1.86 (0.67, 3.05)	0.003	1.08

Note: SUTC = substance use treatment center. Pre-test occurred prior to the training session. Post-test occurred following the training session. Scores range: 0–10.

**Table 3:** Models of organizational demographics and readiness to change as moderators of pre- to post-training knowledge gain (N = 15 SUTCs).

Effect	Estimate	SE	*p*
Organizational Demographics			
Time ^a^	1.869	0.103	<0.001
Number of annual total patient visits	<0.001	<0.001	0.185
Number of annual total patient visits * time	<−0.001	<0.001	0.008
Time ^a^	1.868	0.104	<0.001
Number of annual unique patient visits	<0.001	<0.001	0.466
Number of annual unique patient visits * time	<−0.001	<0.001	0.887
Time ^a^	1.871	0.103	<0.001
Number of full-time employees	0.001	0.001	0.260
Number of full-time employees * time	−0.004	<0.001	<0.001
Organizational Readiness			
Time ^a^	1.869	0.104	<0.001
ORIC resource availability	0.265	0.278	0.342
ORIC resource availability * time	−0.042	0.315	0.893
Time ^a^	1.869	0.104	<0.001
ORIC change efficacy	−0.323	0.396	0.415
ORIC change efficacy * time	0.669	0.306	0.029
Time ^a^	1.869	0.104	<0.001
ORIC change valence	1.469	1.580	0.353
ORIC change valence * time	2.810	1.267	0.027
Time ^a^	1.894	0.103	<0.001
ORIC change commitment	−0.013	0.310	0.966
ORIC change commitment * time	1.224	0.320	<0.001
Time ^a^	1.855	0.103	<0.001
ORIC task knowledge	0.309	0.254	0.224
ORIC task knowledge * time	−0.987	0.268	<0.001
Time ^a^	0.103	18.095	<0.001
ORIC total	0.787	−0.486	0.627
ORIC total * time	0.902	2.904	0.004

Note: ORIC = organizational readiness for implementing change. ^a^ Reference: pre-test. * = interaction term (e.g., A * B denotes the interaction term of A and B).

## Data Availability

Data available on request due to privacy restrictions. The data presented in this study are available on request from the corresponding author. The data are not publicly available due to agreements with funders.
